# The New Reality of Infective Endocarditis: Changes in Patient Demographics and Outcomes in South Carolina

**DOI:** 10.3390/idr17030067

**Published:** 2025-06-11

**Authors:** Grant Garrison, Julie Royer, Max Habicht, Sarah Battle, Hana R. Winders, Kayla Antosz, Anna-Kathryn Burch, Majdi N. Al-Hasan, Julie Ann Justo, Pamela Bailey

**Affiliations:** 1 Department of Medicine, University of Alabama at Birmingham, Birmingham, AL 35233, USA; pggarrison@uabmc.edu; 2 South Carolina Revenue and Fiscal Affairs Office, Columbia, SC 29203, USA; 3 South Carolina Department of Health and Environmental Control, Columbia, SC 29203, USA; 4 School of Medicine, University of South Carolina, Columbia, SC 29203, USA; 5 Division of Infectious Diseases, Department of Medicine, Prisma Health Midlands, Columbia, SC 29203, USAkantosz@mailbox.sc.edu (K.A.); 6 College of Pharmacy, University of South Carolina, Columbia, SC 29203, USA; 7 Prisma Health Children’s Hospitals, Columbia, SC 29203, USA; 8 Dartmouth Hitchcock Medical Center, Bennington, VT 05201, USA

**Keywords:** infective endocarditis, opioid use disorder, population analysis

## Abstract

**Background:** Rising rates of opioid use disorder (OUD), usually via injection, has resulted in younger patients being diagnosed with infective endocarditis (IE), with unique treatment challenges. **Methods:** This retrospective ecological study analyzed hospital discharge and home health records from 2016 to 2022 in South Carolina (SC). Cases of IE with concurrent coding for OUD were identified. Differences in patient demographics, hospital characteristics, length of care days, and charges by OUD status were determined using chi-square or *t*-tests. IE hospitalization rates by OUD status, year, and age group were calculated, and linear regression was used to determine differences by year. **Results:** There were 8601 acute-care hospitalization records for IE from 2016 to 2022 in the SC dataset, of which 1180 (13.7%) had concurrent OUD coding. Statistically significant differences between patients with and without OUD were identified for sex, age group, race, resident rurality, average number of comorbidities, disposition status, and year (all *p* < 0.01). The incidence rate of IE increased from 2.5/100,000 in 2016 to 6.9/100,000 in 2022 in patients aged 36 to 49 years with OUD (*p* = 0.02). Patients with IE and OUD who were discharged home had significantly longer lengths of stay in acute care hospitals (32.9 vs. 15.3 days; *p* < 0.01) and excessive hospital charges ($308,874 vs. $188,862) compared to those without OUD. **Conclusions:** Major changes have occurred in the demographics of IE in SC. The increasing incidence rate of IE in younger adults with OUD coupled with prolonged stays at acute care hospitals pose challenges to the healthcare system that require creative solutions.

## 1. Introduction

The juxtaposition of the opioid epidemic and opioid use disorder (OUD) have long been pertinent to healthcare providers, particularly regarding the complexities of care around infective endocarditis (IE), a rare but serious infection seen on the heart valves [1]. Traditionally, IE has been attributed to older patients and those with prosthetic heart valves or cardiac devices, as described by the Infectious Diseases Society of America guidelines [2]. Injection drug use-related IE has been documented in 7% of a 2021–2022 cohort of persons who inject drugs, with 20% reporting untreated infection symptoms within the preceding three months (Ganesh). IE continues to present challenges leading to high morbidity and mortality, ranking among the top life-threatening infectious syndromes [1].

As a sector of healthcare impacted by the many facets of the opioid epidemic, infectious disease (ID) practitioners have been raising the alarm for an increasing number of younger patients with IE as the opioid epidemic has taken root in America, particularly affecting rural communities [3,4]. Multidisciplinary care including ID, cardiology, and cardiac surgery in patients with IE has been shown to significantly improve patient outcomes [5,6,7,8]. However, many of America’s counties are faced with a lack of access to ID physicians [9]. In rural communities, people are at a median distance of 30.5 km from the nearest ID physician [10], contributing to the disparities in the care provided to patients, increased lengths of hospitalizations, increased patient costs, and, ultimately, differences in patient outcomes [3,4,11]. Due to these disparities in care, the healthcare burden that IE has placed upon healthcare providers and the healthcare system has grown tremendously.

IE is frequently treated with 4–6 weeks of intravenous (IV) antibiotics, which can be administered in hospital or at home if patients are capable. However, there are concerns regarding discharging persons who inject drugs (PWID) home with IV access, which can be tampered with to inject non-prescription drugs [1,2]. Emergency room visits are also common during the antibiotic treatment course after hospital discharge, further increasing healthcare costs to patients who are frequently without healthcare coverage [12]. Often, PWID who have a diagnosis of IE are discharged from the hospital against medical advice at a rate of approximately 10–20 times higher than others, with a 30-day readmission rate, more than double that of all other conditions [13].

In the face of the presumed changes in the epidemiology of IE in South Carolina, the Antimicrobial Stewardship Collaborative of South Carolina aimed to characterize the current demographics of IE in South Carolina, specifically examining the concurrence of OUD in IE hospital admissions to identify PWID and better understand their outcomes and unique treatment barriers. We specifically aimed to focus on rural/urban differences in treatment outcomes, as well as the differences from a non-PWID population.

## 2. Methods

### 2.1. Data Sources and Definitions

At the behest of the Antimicrobial Stewardship Collaborative of South Carolina, the Data Integration and Analysis Division of the South Carolina Revenue and Fiscal Affairs Office queried the all-payer hospital discharge dataset and the all-payer home health dataset; this includes uninsured and self-pay patients. These are administrative data and do not include specific details about what happened during the hospitalization, unless there were codes associated with particular procedures or events in hospital. The Institutional Review Board deemed this project exempted as not human subjects research, and data use approval was obtained from the SC Data Oversight Council. The hospital discharge dataset includes all inpatient hospitalizations from civilian hospitals (approximately 70 hospitals in 2022) within SC, irrespective of payment source, and each patient has a unique identifier in the system to allow linkage of visits. Home health data include services received in an individual’s home provided by a SC home health agency, irrespective of payment source.

A retrospective review of records from 2016 to 2022 identified SC residents with an acute-care inpatient stay with an International Classification of Diseases, 10th revision (ICD-10) code for a diagnosis of IE (Table A1). Patient discharge records indicated one primary diagnosis and up to fourteen secondary diagnosis fields, including IE stays with a concurrent coding for opioid abuse/dependence disorder, OUD, poisoning, or adverse effects by opioids or related substances were identified. Patients less than 18 years of age at the time of the hospital stay were excluded from the analyses. If a patient was discharged from one facility and admitted to another facility on the same day, it was considered a transfer and counted as a single stay. Length of stay (LOS) and charges were summarized across stays, including for transfers and transitions of care, due to frequency of occurrence in the database. Hospital types included acute-care, rehabilitation, specialty hospital care, and long-term acute-care. Skilled nursing home health days counted towards the treatment care measure if initiated within 10 days of discharge. Skilled nursing services were identified by revenue codes beginning with 055. If a patient was transferred, the last stay was utilized for determining the patient disposition status and timing of home health engagement. Rurality was defined by the patient’s zip code and hospital county. Elixhauser comorbidity software (Elixhauser Comorbidity Software Refined for ICD-10-CM Healthcare Cost and Utilization Project (HCUP). January 2025. Agency for Healthcare Research and Quality, Rockville, MD. www.hcup-us.ahrq.gov/toolssoftware/comorbidityicd10/comorbidity_icd10.jsp (accessed on 20 May 2025)) for ICD-10-CM was used to determine comorbidities; Elixhauser Comorbidity Index is a weighted index used to assess a patient’s overall disease burden [14]. Elixhauser comorbidity software creates 38 measures [13]. For this analysis, the valvular disease measure and drug abuse measure were not counted towards number of comorbidities, however ICD codes were used to add frequencies to the number of comorbid conditions. Hospital charges were used, and adjusted for inflation to 2022 dollars using the Gross Domestic Product price index, a tool for adjusting price of goods and services in the United States [15]. We were unable to capture more granular information in this database, such as ID consultation.

### 2.2. Statistical Analyses

Differences in patient demographics and hospital characteristics by OUD status were determined using chi-square tests and *t*-tests for ordinal and continuous variables, respectively, or Wilcoxon–Mann–Whitney tests for non-normally distributed data. Differences in LOS and charges by OUD status and disposition status were determined using *t*-tests. Linear regression was used to determine differences in inpatient stay rates by age group and OUD status over time. To address multiple testing, *p*-values were adjusted using the Holm-Bonferroni method. SAS 9.4 was used in the analyses (SAS Institute Inc., Cary, NC, USA).

To compare SC IE inpatient stay rates to national and regional rates, cross-border acute-care inpatient stays from Georgia and North Carolina for South Carolina residents were added to SC acute-care facility discharge data. There are SC citizens who were hospitalized in these neighboring states. Inpatient stays for endocarditis and endocardial disease were identified using the Agency for Healthcare Research and Quality (AHRQ) Healthcare Cost and Utilization Project (HCUP) clinical classification software refined (CCSR) for ICD-10 diagnoses, using CIR004 for IE [16]. Postcensal SC population estimates were used to calculate a state rate [17]. National, South Atlantic, and South Region endocarditis and endocardial disease inpatient stay rates were identified using the HCUPnet data query tool; HCUPnet data at the time of the study was available through 2020 [18].

## 3. Results

There were 8601 acute-care inpatient stay records for IE from 2016 to 2022 in the South Carolina hospital discharge dataset, and a subset of 1180 (13.7%) patients for whom there was concurrent coding for OUD. After considering transfers, 6634 inpatient stays without concurrent coding for OUD and 1047 inpatient stays with coding for OUD remained. Transfers included ones to another general hospital for acute inpatient care or transfers to another facility type (SNFs, hospice, inpatient rehabilitation, and long-term acute-care facilities). Patient demographics and hospital stay characteristics by OUD status are shown in Table 1. Compared to patients without OUD, those with OUD were more likely to be younger in age, have female sex, white race, and urban residence. Patients with OUD were more likely to be discharged against medical advice than those without OUD. The number of treatment days for each inpatient IE stay—the sum of the days in the hospital and skilled nursing care days after discharge—was further stratified by patients discharged home and those who left against medical advice as shown in Table 2. Patients with OUD who were discharged home had longer hospital lengths of stay and more healthcare charges than those without OUD.

**Figure 1:**
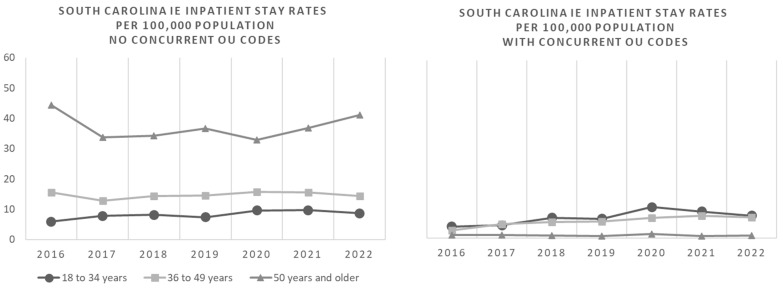
South Carolina infective endocarditis (IE) inpatient stay incidence rates per 100,000 population by year, age group, and by concurrent coding status for opioid abuse/dependence disorder, opioid use, poisoning, or adverse effects by opioids or related substances (OU).

**Figure 2:**
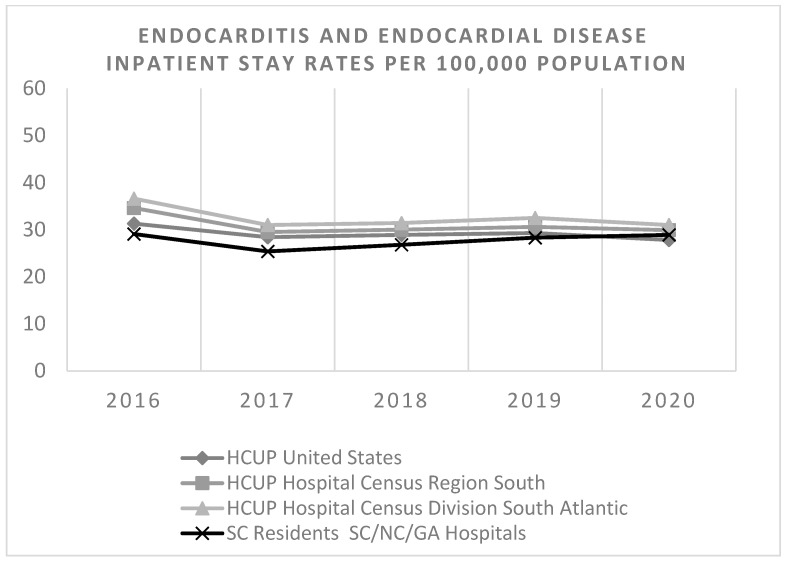
Incidence rate of inpatient stays related to endocarditis and endocardial disease per 100,000 for the United States, South Region, South Atlantic Region, and South Carolina. The South Atlantic Division includes Delaware, Maryland, District of Columbia, Virginia, West Virginia, North Carolina, South Carolina, Georgia, and Florida. The South Region contains three divisions: South Atlantic, East South Central (Alabama, Kentucky, Mississippi, Tennessee), and West South Central (Arkansas, Louisiana, Oklahoma, Texas).

Figure 1 shows IE inpatient stay rates per 100,000 South Carolinians by OUD status over the course of the study period through 2022. There was a significant increase in the incidence rate of IE hospitalizations between 2016 and 2022 in patients aged 36 to 49 years with OUD (adjusted *p*-value = 0.02). There were no significant changes in the incidence rates of IE hospitalizations during the study period in patients with OUD in other age groups, in patients with OUD, or in those without OUD. National, regional, and SC incidence rates of inpatient stays related to endocarditis and endocardial disease per 100,000 through 2020 are comparable as shown in Figure 2.

## 4. Discussion

This study demonstrates significant differences in demographics, clinical characteristics, outcomes, and healthcare costs between patients with and without OUD hospitalized for IE in South Carolina, demonstrating the importance of rallying around the opioid crisis as an ongoing public health crisis. Over 70% of patients with IE and OUD were younger than 40 years old, compared to only 14% of those without OUD. The almost 3-fold increase in the incidence rate of IE hospitalization in those with OUD aged 36 to 49 years between 2016 and 2022 in South Carolina is alarming, and can seriously overstretch healthcare systems dealing with these serious infections. This pattern seems to be consistent with other geographical locations in the United States, as well as with COVID-19-related spikes during the pandemic [19,20,21]. The prolonged length of stay in acute care hospitals by over 2 weeks and excessive hospital charges by over $100,000 in patients with IE and OUD compared to those without OUD represent additional burdens on an already overstretched healthcare system that traditionally keeps these patients in hospital for the duration of hospitalization instead of addressing more creative ways to manage this complex condition. The additional complication of very high rates of against-medical-advice discharges emphasizes the challenges inherent in this population as well. These unique changes in the epidemiology and outcomes of IE support the need for a specific focus in the management guidelines of IE on this high-risk population.

Along with the changing demographics that we are seeing with IE, the healthcare setting these patients are seen in has created the need for new approaches to treatment. Many of the patients presenting from rural zip codes are faced with socioeconomic challenges that create obstacles in access to care. With up to 80% of United States counties not having access to an ID physician, these patients are frequently transferred to an acute care facility for specialty services or managed by hospitalist physicians without specific training in the nuances of IE [9]. This is particularly concerning in this study considering South Carolina and fits with the national assessment of a majority of counties, particularly in rural areas, not having access to ID clinicians, though we were not able to assess ID consultation in this dataset. Lack of access to ID-trained clinicians leads to significant variations in treatment approaches, and poor patient outcomes are a byproduct of this [7,8]. Infectious diseases consultations improve outcomes in IE; specifically, they improve management, provide better clinical outcomes, and reduce embolic events [8]. If clinical guidelines that can be followed by any provider are updated to address the specific needs of this population, disparities in outcomes may be less impacted by needing to see an ID provider.

Notably, the complexities involved in co-managing OUD-associated endocarditis are compounded by critical social and medical comorbidities prior to the hospitalization, and new impairments are identified during these hospitalizations which are insufficiently managed in the current healthcare system [22]. Both providers and patients see the potential prolonged hospitalization as an opportunity for addressing the complex psychosocial needs including addiction care, but the lack of overall longitudinal care contributes to overall poor health outcomes; therefore, innovative care solutions are required to address systematic barriers [22,23]. Additionally, psychosocial issues such as homelessness, access to food and clothing and other markers of subsistence, justice system involvement, and depression or anxiety, as well as post-traumatic stress disorder, complicate OUD-related IE, and addressing these issues is critical to ensure good outcomes in patients’ care [24,25]. The social and medical comorbidities are intertwined, and hospitals must develop treatment teams capable of addressing these needs, with social work and addiction management playing prominent roles.

Just as treatment approaches vary, the length of hospitalization and total charges vary dramatically for patients with a concurrent diagnosis of OUD, as demonstrated in Table 2. Our study confirms the work performed in a prior study showing younger patients with IE are more likely to use opioids, with significantly increased ICU and hospital LOS, although in this study we are only able to examine acute hospital care without the refinement to ICU [3]. As a direct result of these significantly longer hospitalizations, the total charges, which may differ from actual costs to patients, that this subset of patients are facing have shown an increase of >$100,000 (Table 2). Length of hospitalization can easily be attributed to the complications of IE and OUD, including septic emboli to the lungs or other organs, or the hesitance of providers to discharge patients with home intravenous antibiotics due to the concern for tampering or improper use of PICC lines in PWID.

Innovative care includes moving towards oral IE treatment in patients with (and without) OUD, regardless of concerns about intravenous lines leading to potential abuse. Nine percent of outpatient parenteral antimicrobial therapy courses are complicated by vascular access complications requiring clinical interventions, and injection drug use had a 3-fold-increased incidence of said complications [26]. Additionally, there are stigma-related inequities in care, particularly around PWID and peripherally inserted central catheters (PICC), and limitations on discharge destination [22,27]. Oral antibiotics have been demonstrated to have comparable efficacy to intravenous therapy in patients with complicated bloodstream infections, including infective endocarditis [28]. In fact, one of the earliest studies on this topic specifically examined right-sided endocarditis in PWID [26]. In the consensus management guidelines, oral transitional therapy is considered at least as effective as intravenous therapy for treatment of IE [29]. Left-sided endocarditis in stable patients were able to change to oral antibiotics to complete their course with non-inferiority shown compared to intravenous antibiotics [30]. Additionally, long-acting lipoglycopeptides (e.g., dalbavancin) have been shown to reduce LOS and improve cost-effectiveness by using them to discharge patients earlier while still having the long-acting lipoglycopeptide in their system [31]. There is evidence that transition from IV to oral antibiotic therapy is superior to incomplete intravenous antibiotic treatment [1]. Considering the findings in this population-based study, it is worthwhile to educate providers on these studies and modalities that have sufficient clinical data to support their use, even if guidelines have yet to be written which incorporate these studies.

Limitations of this study include the utilization of administrative data; ICD-10 codes may be inaccurate or poorly characterized for the individual patient or overestimate readmission for the same patient, leading to potentially inaccurate data. We attempted to gather data about infectious-diseases-clinician involvement and specific comorbid conditions, but were unable to obtain that information from the database. Additionally, population level data like this can lead to ecologic (or population) fallacy, generating inferences about an individual from the group to which they belong. This is complicated by potential confounding factors that may allow patients to have different levels of access to care, such as socioeconomic status. Nuances such as device-related IE, co-stimulant use, particularly considering we used the surrogate ICD codes regardless, and phase of the opioid crisis cannot be accommodated with the lack of specificity in this type of dataset, limiting generalizations that can be made with these analyses.

## 5. Conclusions

Administrative data show that younger patients are a growing burden of the IE cases, with expensive hospital stays and longer LOS if potential PWID. This is a significant burden in the healthcare system and an ongoing public health crisis that demands action with newer guidelines needed to address this changing epidemiology, particularly around accommodating alternative treatment strategies in order to improve the outcomes of this population. These guidelines need to be broadly applicable across all care settings to allow disparities in care to resolve and allow all patients to have excellent clinical outcomes.

## Figures and Tables

**Table 1:** Infective endocarditis inpatient stay characteristics by concurrent coding status for opioid abuse/dependence disorder, opioid use, poisoning, or adverse effects by opioids or related substances.

	Infective Endocarditis Without Concurrent Opioid Use, Abuse, or Dependence Codes		Infective Endocarditis with Concurrent Opioid Use, Abuse, or Dependence Codes		
	Number	Percentage	Number	Percentage	Adjusted *p*-Value *
Inpatient Stays	6634	100.0%	1047	100.0%	
Unique Patients	5441	−	721	−	
Sex					
Males	3877	58.4%	472	45.1%	
Females	2757	41.6%	575	54.9%	<0.01
Race					
White	4578	69.0%	952	90.9%	
Black	1880	28.3%	68	6.5%	
Other/Missing	176	2.7%	27	2.6%	<0.01
Age Group					
18 to 35	707	10.7%	599	57.2%	
36 to 49	912	13.7%	328	31.3%	
50 and older	5015	75.6%	120	11.5%	<0.01
Resident Rurality **					
Urban	4983	75.3%	839	80.9%	
Rural	1632	24.7%	198	19.1%	<0.01
Average Number of Comorbid Conditions	3.5		2.3		<0.01
Study Year					
2016	1018	15.4%	84	8.0%	
2017	836	12.6%	110	10.5%	
2018	880	13.3%	144	13.8%	
2019	936	14.1%	141	13.5%	
2020	898	13.5%	209	20.0%	
2021	989	14.9%	188	18.0%	
2022	1077	16.2%	171	16.3%	<0.01
Patient Disposition Status					
Expired/Hospice	1476	22.3%	80	7.6%	
Left Against Medical Advice	271	4.1%	340	32.5%	
Home/Home with Home Health	3484	52.5%	533	50.9%	
Other ***	1403	21.2%	94	9.0%	<0.01
Presenting Hospital Bed Size					
Less than 100 Beds	630	9.5%	96	9.2%	
100–299 Beds	2638	39.8%	425	40.6%	
300 or more Beds	3366	50.7%	526	50.2%	0.8597

* Differences by OU status were determined using chi-square tests, except for average number of comorbid conditions. *T*-test procedure was used to compare number of comorbidities. *p*-values adjusted for multiple comparisons using Holm-Bonferroni method. ** 29 inpatient stays missing information for rurality determination. Centers for Medicare and Medicaid Services’ ambulance rural and super-rural zip code assignments were used to determine rural category. *** Other includes transfers to out-of-state acute care facilities, federal facilities, psychiatric hospitals, skilled nursing facilities and intermediate care facilities, nursing homes, planned readmissions, and court/law enforcement.

**Table 2:** Infective endocarditis length of hospital stay in days, home health days, and total charges by patient discharge status and by concurrent coding status for opioid abuse/dependence disorder, opioid use, poisoning, or adverse effects by opioids or related substances.

	Infective Endocarditis Without Concurrent Opioid Use, Abuse, or Dependence Codes			Infective Endocarditis with Concurrent Opioid Use, Abuse, or Dependence Codes			
	N	Mean	(95% CL Mean)	N	Mean	(95% CL Mean)	Adjusted *p*-Value
*Where patient discharged home or home with home health*							
Length of Stay in Days							
Acute Hospital Care	3484	15.3	(14.6, 15.9)	533	32.9	(31.1, 34.7)	<0.01
Specialty Hospital Care	202	25.1	(22.6, 27.5)	33	29.9	(25.6, 34.2)	0.41
Home healthcare	1277	48.2	(46.6, 49.9)	37	45.4	(34.4, 56.5)	0.89
Sum Hospital and Home Health	3484	34.4	(33.2, 35.6)	533	37.9	(35.7, 40.2)	0.18
Total Charges *							
Acute Hospital Care	3484	$188,862	($180,367, $197,356)	533	$308,874	($284,052, $333,696)	<0.01
Specialty Hospital Care	202	$118,826	($105,266, $132,386)	33	$187,539	($127,388, $247,689)	<0.01
Home healthcare	1277	$3427	($3263, $3591)	37	$2357	($1587, $3127)	0.18
Sum Hospital and Home Health	3484	$197,007	($188,292, $205,722)	533	$320,649	($294,338, $346,960)	<0.01
*Where patient left against medical advice*							
Length of Stay in Days							
Acute Hospital Care	271	15.2	(13.0, 17.4)	340	14.2	(12.7, 15.7)	0.89
Total Charges *							
Acute Hospital Care	271	$160,727	($134,468, $186,987)	340	$134,770	($116,957, $152,583)	0.39

* Total charges adjusted for inflation. *p*-values adjusted for multiple comparisons using Holm–Bonferroni method.

## Data Availability

Data were obtained through the South Carolina Revenue and Fiscal Affairs office. The datasets used and/or analyzed during the current study are available from the corresponding author on reasonable request.

## Appendix A

**Table A1:** ICD-10 codes used to define infective endocarditis.

ICD-10-CM Code	ICD-10-CM Code Description
A3282	Listerial endocarditis
A3951	Meningococcal endocarditis
A5203	Syphilitic endocarditis
A5483	Gonococcal heart infection
B3321	Viral endocarditis
B376	Candidal endocarditis
I011	Acute rheumatic endocarditis
I330	Acute and subacute infective endocarditis
I339	Acute and subacute endocarditis, unspecified
I38	Endocarditis, valve unspecified
I39	Endocarditis and heart valve disorders in diseases classified elsewhere
M3211	Endocarditis in systemic lupus erythematosus
F111 *	Opioid abuse
F112 *	Opioid dependence
F119 *	Opioid use
T400X *	Poisoning by, adverse effect of and underdosing of opium
T401X *	Poisoning by and adverse effect of heroin
T402X *	Poisoning by, adverse effect of and underdosing of other opioids
T403X *	Poisoning by, adverse effect of and underdosing of methadone
T404 *	Poisoning by, adverse effect of and underdosing of synthetic narcotics
T507X *	Poisoning by, adverse effect of and underdosing of analeptics and opioid receptor antagonists

* Parent code. All codes below parent code were used to define opioid abuse/dependence disorder, opioid use, or poisoning or adverse effects by opioids or related substances.
